# Physiological and prognostic differences between types of exercise stress echocardiography for functional mitral regurgitation

**DOI:** 10.1136/openhrt-2021-001583

**Published:** 2021-04-22

**Authors:** Nobuyuki Kagiyama, Misako Toki, Takuya Yuri, Shingo Aritaka, Akihiro Hayashida, Partho P Sengupta, Kiyoshi Yoshida

**Affiliations:** 1 Department of Cardiology, Sakakibara Heart Institute of Okayama, Okayama, Japan; 2 Department of Cardiovascular Biology and Medicine, Juntendo University, Tokyo, Japan; 3 Department of Clinical Laboratory, Sakakibara Heart Institute of Okayama, Okayama, Japan; 4 Heart and Vascular Institute, West Virginia University, Morgantown, West Virginia, USA

**Keywords:** heart failure, echocardiography, mitral valve insufficiency

## Abstract

**Objective:**

Secondary mitral regurgitation (MR) demonstrates dynamic change during exercise. This prospective observational study aimed to compare exercise stress echocardiography (ESE) where handgrip exercise (handgrip-ESE) or semisupine ergometer exercise was performed (ergometer-ESE) for patients with secondary MR.

**Methods:**

Handgrip-ESE and symptom-limited ergometer-ESE were performed for 53 patients (median age (IQR): 68 (58–78) years; 70% male) on the same day. Baseline global longitudinal strain (GLS) was 9.2% (6.0%–14.0%) and MR volume was 20 (14–26) mL. All-cause death and cardiac hospitalisation were tracked for median 439 (101–507) days.

**Results:**

Handgrip-ESE induced slightly but significantly greater degrees of MR increase (median one grade increase; p<0.001) than ergometer-ESE, although the changes in other parameters, including GLS (+1.1% vs −0.6%, p<0.001), were significantly smaller. Correlations between the two examinations with respect to the changes in the echocardiographic parameters were weak. Kaplan-Meier analyses revealed poor improvement in GLS during ergometer-ESE, but not the change in MR, was associated with adverse events (p=0.0065). No echocardiographic change observed during handgrip-ESE was prognostic. After adjusting for a clinical risk score, GLS changes during ergometer-ESE remained significant in predicting the adverse events (HR 0.39, p=0.03) A subgroup analysis in patients with moderate or greater MR at baseline (n=27) showed the same results as in the entire cohort.

**Conclusions:**

The physiological and prognostic implications of handgrip-ESE and ergometer-ESE findings significantly differ in patients with left ventricular dysfunction and secondary MR. The type of exercise to be performed in ESE should be carefully selected.

**Key questions**

**What is already known about this subject?**

- Studies have reported that ergometer-exercise stress echocardiography is useful for prognostic assessment in patients with mitral regurgitation. Since ergometer-exercise is often not feasible for elderly and frail patients, doctors sometimes select handgrip exercise as an alternative. However, it has been unknown that handgrip-exercise stress echocardiography is equivalent to ergometer exercise.

**What does this study add?**

- Our study clearly showed that although handgrip-exercise stress echocardiography induces similar degree of mitral regurgitation increase, its physiological and prognostic meaning is different from what observed in ergometer-exercise stress echocardiography.

**How might this impact on clinical practice?**

- Our study raises a caution about handgrip-exercise stress echocardiography as an alternative to ergometer exercise.

## Introduction

Secondary mitral regurgitation (MR) is a common complication observed in approximately half of patients with heart failure with left ventricular (LV) dysfunction,^1–4^ and it is associated with a higher incidence of adverse events.^5^ As a recent randomised controlled trial reported that percutaneous intervention for secondary MR may improve the clinical outcomes in appropriately selected populations, the importance of detailed and accurate assessment of secondary MR is being acknowledged.^6^ The dynamic nature of the severity of MR is one of the major difficulties encountered in evaluation of secondary MR. Since the severity of secondary MR is determined by the degree of mitral leaflet tethering caused by papillary muscle dislocation, the degree of secondary MR varies significantly depending on the LV volume and systolic function, which change often based on the preload and afterload volumes.^7–9^

Exercise stress echocardiography (ESE) for evaluation of valvular heart disease is a well-known examination, which allows real-time evaluation of the dynamic changes in the severity of valvular regurgitation and LV function. Previous studies have reported that quantitative assessment of MR and LV function during ergometer exercise is reproducible and has prognostic significance for primary MR.^8 10 11^ However, prognostic data of ESE for patients with secondary MR are sparse, although exercise intolerance is a major predictor of adverse events in heart failure.^12 13^ In clinical practice, patients with secondary MR, especially those who are indicated for percutaneous mitral valve repair, are often old, frail and intolerant of strenuous exercises.^14^ Hence, handgrip exercise often serves as an alternative in stress tests for such frail patients.^15^ Although the increase in the severity of secondary MR during handgrip exercise is frequently observed in individual cases,^15^ the physiological consequences of handgrip exercise, an isometric exercise, are different from those of common isotonic exercises, like treadmill and ergometer exercises. Thus, this study aimed to investigate (1) the physiological differences between ESE where handgrip exercise was performed (handgrip-ESE) and ESE where semisupine ergometer exercise was performed (ergometer-ESE) in patients with heart failure and secondary MR, and (2) their prognostic implications in such a population.

## Methods

### Study population

We conducted a prospective single-centre observational study that included patients who underwent ESE in our hospital from October 2015 to December 2016. Consecutive patients with (1) LV ejection fraction (LVEF) <40% and secondary MR. Based on the European Society of Cardiology and European Association for Cardiothoracic Surgery guidelines,^16 17^ severe MR was defined by comprehensive approach with cut-off values of MR volume >30 mL and effective regurgitant orifice >0.2 cm^2^; (2) absence of degenerative changes in the mitral leaflet, such as myxomatous change, billowing, prolapse, heavy calcification, infective endocarditis, anomaly and perforation; (3) absence of active ischaemic disease or significant coronary stenosis; and (4) tolerance to at least 25–50 W of ergometer exercise were enrolled.

All patients underwent laboratory tests for the assessment of the creatinine and B-type natriuretic peptide levels. The Meta-Analysis Global Group In Chronic heart failure (MAGGIC) mortality risk score was calculated, as described previously.^18^ All-cause death and cardiac hospitalisation were tracked via medical chart or telephone interview. The study protocols complied with the guidelines of the Declaration of Helsinki and were approved by the institutional review board. All patients signed the written informed consent forms.

### Stress echocardiography

Baseline echocardiography was performed in the supine position at rest. Handgrip-ESE was subsequently performed in the supine position. The patients were asked to grip a dynamometer with half their maximum strength for 8 min using either hand, and echocardiographic images were acquired in the last 3 min of the exercise. The dynamometer indicates a real-time grip strength, and dedicated medical staff was observing the grip strength, confirming that the patient was appropriately keeping the grip strength during the exercise. After the handgrip-ESE, a minimal 30 min interval was ensured before the patients underwent symptom-limited ergometer-ESE. The workload was initially 25 W and was then increased by 25 W every 3 min. Echocardiographic images were acquired at the final workload by an experienced sonographer in the last 1–2 min of the test, as described previously.^19^ During these exercise protocols, the blood pressure and 12-lead ECG were monitored. All echocardiographic examinations were performed using commercially available equipment (Aplio Artida, Canon Medical Systems Corporation, Tochigi, Japan), which was maintained in accordance with the guidelines.^20 21^ The LVEF was calculated with the 2D method of disks using the apical 2-chamber and 4-chamber views. The severity of MR was graded using a multiparametric approach, including the ratio of the MR area to the left atrial (LA) area and proximal isovelocity surface area methods, as recommended in the guidelines.^22^ The systolic pulmonary artery pressure (PAP) was calculated by adding 10 mm Hg to pressure gradient between the right atrium and right ventricle.^10^ The speckle tracking strain was measured using vendor-independent software (Image Arena, TomTec, Germany), and global longitudinal strain (GLS) was calculated as an average of the 18 segments derived from the three standard apical views.^23^ In the present study, the GLS was expressed in absolute values to avoid confusion, as proposed previously.^24 25^ Echocardiographic movies were stored for three heartbeats, and all analyses, including speckle tracking, were performed offline over multiple beats, as required. A trained sonographer or a cardiologist, who was blinded to the patients’ clinical information and outcomes, analysed the images according to the guidelines.^23^

### Statistical analysis

The data are presented as the median (IQR) for the continuous variables and as the frequency (%) for the categorical variables. Group differences were evaluated using the Mann-Whitney U and Χ^2^ or Fisher’s exact tests for the continuous and categorical variables, respectively. Differences between the continuous parameters obtained at peak exercise and the baseline values were compared using paired t-tests; the differences noted between these changes during the two tests were also evaluated. The MR grade was compared using the Wilcoxon signed-rank test with relevelling of the MR grade to ordinal variables (mild=1, moderate=2, moderate-to-severe=3 and severe=4). The Pearson’s correlation tests were used to evaluate the relationship of between the changes in the parameters during handgrip-ESE and those during ergometer-ESE. Weighted kappa was used to test the agreement in the MR grade during the two examinations. Moreover, we evaluated the parameters associated with the changes in MR volume and GLS to assess the mechanisms underlying these changes during the two types of exercise using these tests. For survival analysis, the patients were divided according to the median values of the parameters of interest, and the Kaplan-Meier curve analysis, log-rank test and multivariable Cox proportional hazard models were used. As a sensitivity analysis, we repeated all the analyses with a subgroup of patients who had moderate or greater MR.

All statistical analyses were performed with R V.3.5.2 (The R Foundation for Statistical Computing, Vienna, Austria). A two-tailed p<0.05 indicated statistical significance.

### Patient and public involvement

This study was completed without patient involvement.

## Results

### Study cohort

After excluding one patient due to images of insufficient quality, 106 stress tests were performed for 53 patients whose image quality was sufficient for quantitative analysis. The exercise was standardised and submaximal for all. Table 1 summarises the patient characteristics. The median age was 68 (58–78) years, and 69.8% of the patients were male, while 45.3% showed ischaemic aetiology without residual stenosis or active ischaemic episodes. Over one-third of the patients showed significant symptoms of heart failure with New York Heart Association (NYHA) grade III, and the MAGGIC risk score was overall high (30 (23–33)). There was no patient with NYHA IV. At baseline, most patients showed very low LVEF (26% (16%–35%)) and GLS (9.2% (6.0%–14.0%)). The severity of secondary MR was mild in 43%, moderate in 42%, and moderate-to-severe or severe in 15% of the patients. Median MR volume was 20 (14–26) mL and MR–LA area ratio was 0.12 (0.10–0.17).

**Table 1:** Patient characteristics

Parameter	
Age, years old	68 (58–78)
Male, n (%)	37 (69.8)
Body mass index (kg/m^2^)	23.0 (20.1–24.7)
Systolic blood pressure, mm Hg	110 (99–124)
Diastolic blood pressure, mm Hg	69 (64–75)
Heart rate, /min	69 (60–77)
Medical history	
Ischaemic cardiomyopathy, n (%)	24 (45.3)
COPD, n (%)	18 (34.0)
Diabetes, n (%)	31 (58.5)
Heart failure severity	
NYHA class ≥III, n (%)	18 (34.0)
B-type natriuretic peptide, pg/mL	284 (186–520)
MAGGIC score	30 (23–33)
Echocardiography	
Interventricular septum, mm	10 (8–11)
LV diastolic diameter, mm	59 (52–66)
LV systolic diameter, mm	50 (42–60)
Left atrial diameter, mm	44 (39–49)
LV ejection fraction, %	26 (16–35)
Mitral E velocity, cm/s	81 (66–102)
Tissue Doppler e', cm/s	4.6 (3.5–5.7)
E/e'	16.0 (12.3–24.6)
Right atrial pressure, mm Hg	8 (3–8)
Systolic PAP, mm Hg	33 (29–39)
GLS, % (absolute value)	9.2 (6.0–14.0)
MR volume, mL	20 (14–26)
Mitral EROA, cm^2^	0.12 (0.10–0.17)
MR/LA area ratio	0.21 (0.12–0.27)
Mitral valve tenting height, mm	9.3 (8.0–10.9)
MR grade, n (%)	
Mild	26 (49.1)
Moderate	19 (35.8)
Moderate-to-severe	7 (13.2)
Severe	1 (1.9)

COPD, chronic obstructive pulmonary disease; EROA, effective regurgitant orifice; GLS, global longitudinal strain; LA, left atrium; LV, left ventricular; MAGGIC, Meta-Analysis Global Group In Chronic heart failure; MR, mitral regurgitation; NYHA, New York Heart Association; PAP, pulmonary artery pressure.

### Exercise stress echocardiography

All patients underwent handgrip-ESE and ergometer-ESE without any complications. None of the patients developed any symptoms of ischaemia, such as chest pain, induced wall motion abnormality or ECG changes. The median workload during ergometer-ESE was 50 (50–50) W. The significant increase in heart rate (+40 and +10/min by ergometer-ESE and handgrip-ESE, respectively; isotonic exercise does not usually increase heart rate much) and blood pressure (+38 and +25 mm Hg, respectively) by the exercises indicates that the exercises were properly performed, and the amount of workload was significant. MR volume assessment was feasible for all patients, except for one patient due to insufficient image quality for GLS analysis during ergometer-ESE. The assessment of the tricuspid valve regurgitation peak velocity was not feasible for seven patients due to minimum regurgitation, and these patients were excluded from analyses using PAP.

**Figure 1:**
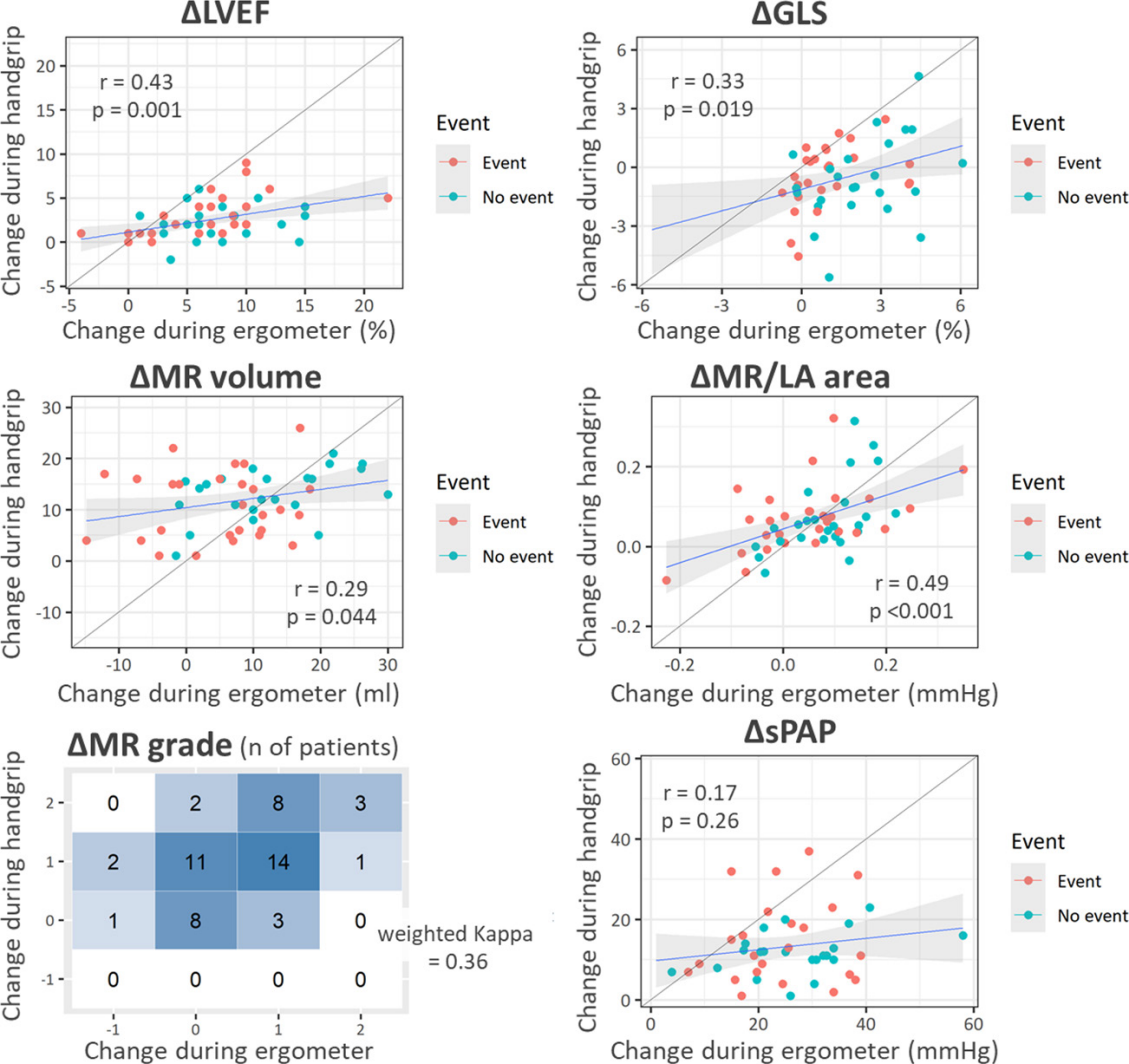
Correlation between the changes during handgrip-ESE and ergometer-ESE. In each panel, the x and y axes show the changes in the parameter during ergometer-ESE and handgrip-ESE. All parameters showed significant but only mild-to-moderate correlation between handgrip-ESE and ergometer-ESE. ESE, exercise stress echocardiography; GLS, global longitudinal strain; LA, left atrium; LVEF, left ventricular ejection fraction; MR, mitral regurgitation; sPAP, systolic pulmonary artery pressure.

Table 2 shows the changes in the vital signs and echocardiographic parameters during handgrip-ESE and ergometer-ESE. As shown in table 2, both the handgrip-ESE and ergometer-ESE showed significant degree of increase in MR, whereas the increase during handgrip-ESE was slightly but significantly greater (MR volume,+12 (6–16) mL vs +9 (1–15) mL, p=0.013, and MR grade +1 (1–1) vs +1 (0–1), p<0.001). In contrast, the changes in all other parameters, except for E/e’ (p=0.26), were significantly greater during ergometer-ESE than during handgrip-ESE (p<0.001). Specifically, the GLS (−0.6% (−1.3% to +0.5%), p=0.052 vs baseline) and LV stroke volume (−1 mL (−7 to +3 mL), p=0.14) did not increase significantly during handgrip-ESE; rather, these values tended to decrease. Figure 1 shows the relationship between the changes in the echocardiographic parameters during handgrip-ESE and ergometer-ESE, and online supplemental figure 1 shows the Bland Altman plots for them. The correlation between the changes during handgrip-ESE and ergometer-ESE was weak or moderate at most (r=0.29–0.49), and the changes in MR grading during the two examinations showed only moderate degree of agreement (weighted kappa=0.36; the same degree of change in 47%, more increase by handgrip in 45% and more increase by ergometer in 8%).

10.1136/openhrt-2021-001583.supp1 Supplementary data

**Table 2:** Change by each exercise

Parameter	Ergometer-ESE	Handgrip-ESE	P value (ergometer-ESE vs handgrip-ESE)
Δ Heart rate, /min	+40 (+25 to +52)**	+10 (5 to 16)**	<0.001
Δ Systolic blood pressure, mm Hg	+38 (+22 to +56)**	+25 (16 to 40)**	<0.001
Δ LV ejection fraction, %	+6 (+3 to +9)**	+2 (1 to 4)**	<0.001
Δ Stroke volume, mL	+11 (+4 to +19)**	−1 (−7 to 3)	<0.001
Δ E/e'	+1.0 (−1.9 to 5.3)	+1.6 (–1 to 3.5)*	0.26
Δ Right atrial pressure, mm Hg	+5 (±0 to +5)**	±0 (±0 to ±0)*	<0.001
Δ Systolic PAP, mm Hg	+25 (+19 to +33)**	+11 (+7 to +18)**	<0.001
Δ GLS, % (absolute value)	+1.1 (0.3 to 2.9) **	−0.6 (–1.3 to 0.5)	<0.001
Δ MR volume, mL	+9 (+1 to +15)**	+12 (+6 to +16)**	0.013
Δ MR/LA area ratio	+0.07 (−0.01 to +0.13)**	+0.06 (+0.02 to +0.10)**	0.59
Δ MR grade	+1 (+0 to +1)**	+1 (+1 to +1)**	<0.001

*P<0.05 vs baseline; **p<0.001 vs baseline. Paired t-tests were used for comparison except for MR grade which was compared using Wilcoxon signed-rank test.

ESE, exercise stress echocardiography; GLS, global longitudinal strain; LA, left atrium; LV, left ventricular; MR, mitral regurgitation; PAP, pulmonary artery pressure.

Table 3 summarises the parameters that were significantly correlated with the increase in the GLS and/or MR grade. The parameters associated with the changes in the GLS and/or MR during ergometer-ESE and handgrip-ESE were completely different, suggesting different underlying mechanisms.

**Table 3:** Parameters correlated with changes in GLS and MR volume

Exercise type	Correlation with Δ GLS			Correlation with Δ MR volume		
	Parameter	r (95% CI)	P value	Parameter	r (95% CI)	P value
Ergometer-ESE	Δ e'	0.36 (0.08 to 0.58)	0.012	Δ E/e'	0.36 (0.09 to 0.58)	0.010
	Δ E	0.35 ([0.07 to 0.57)	0.015	Δ E	0.32 (0.04 to 0.54)	0.024
	Δ sPAP	0.32 (0.01 to 0.57)	0.040	baseline ESV	−0.28 (−0.51 to −0.01)	0.040
	baseline sPAP	−0.34 (−0.58 to −0.04)	0.027	baseline E/e'	−0.35 (−0.56 to −0.08)	0.011
	baseline E	−0.36 (−0.58 to −0.09)	0.010	baseline E	−0.35 (−0.57 to −0.09)	0.010
	baseline E/A	−0.44 (-0.68 to -0.12)	0.009	baseline RAP	−0.38 (−0.59 to −0.12)	0.006
				baseline E/A	−0.43 (−0.66 to −0.11)	0.011
				baseline sPAP	−0.50 (−0.69 to −0.24)	<0.001
Handgrip-ESE	baseline E/A	−0.37(−0.63 to −0.03)	0.033	Δ diastolic BP	0.32 (0.05 to 0.55)	0.021
	Δ diastolic BP	−0.44 (−0.64 to −0.19)	0.001			

BP, blood pressure; ESE, exercise stress echocardiography; ESV, end-systolic volume; GLS, global longitudinal strain; MR, mitral regurgitation; RAP, right atrial pressure; sPAP, systolic pulmonary artery pressure.

### Prognostic implication of each examination

**Figure 2:**
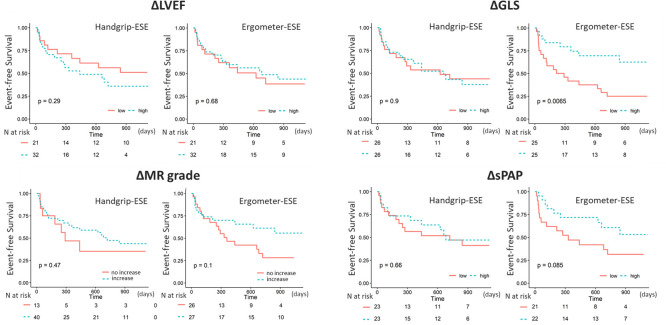
Association of the changes during exercise with clinical outcomes. The Kaplan-Meier curves in each panel show the association of the changes in the parameter during handgrip-ESE (left side) and ergometer-ESE (right side) with all-cause mortality and cardiac hospitalisation. Poor GLS improvement during ergometer-ESE, but not during handgrip-ESE, was significantly associated with a lower adverse event rate. The changes in the LVEF, MR grade and sPAP during either handgrip-ESE or ergometer-ESE did not have prognostic implications for the patients. ESE, exercise stress echocardiography; GLS, global longitudinal strain; LVEF, left ventricular ejection fraction; MR, mitral regurgitation; sPAP, systolic pulmonary artery pressure.

During the median follow-up period of median 439 (101–507) days, 28 patients experienced adverse cardiac events, including deaths of seven patients. The Kaplan-Meier curves, with cut-off values of median values as mentioned above, are shown in figure 2. Only poor improvement in the GLS (below median) during ergometer-ESE, but not the change in MR or other parameters, was associated with a higher incidence of adverse events. None of the echocardiographic changes observed during handgrip-ESE had prognostic implications for the patients. Even after adjusting for the MAGGIC risk score, a well-established robust risk score for heart failure, a significant increase in the GLS during ergometer-ESE (adjusted HR 0.39, 95% CI 0.16 to 0.91, p=0.030) was noted (table 4).

**Table 4:** Cox proportional hazard models for adverse events

Exercise type	Parameter	Univariable		Adjusted by MAGGIC score	
		HR (95% CI)	P value	HR (95% CI)	P value
Ergometer-ESE	High Δ LVEF	0.85 (0.40 to 1.81)	0.68	0.85 (0.40 to 1.80)	0.67
	**High Δ GLS**	**0.33 (0.14 to 0.77**)	**0.010**	**0.39 (0.16 to 0.91**)	**0.030**
	Increased MR grade	0.52 (0.25 to 1.11)	0.09	0.55 (0.26 to 1.18)	0.13
	High Δ sPAP	0.65 (0.28 to 1.48)	0.31	0.65 (0.29 to 1.49)	0.31
Handgrip-ESE	High Δ LVEF	1.52 (0.70 to 3.31)	0.29	1.45 (0.67 to 3.17)	0.35
	High Δ GLS	1.05 (0.50 to 2.20)	0.90	1.04 (0.50 to 2.20)	0.91
	Increased MR grade	0.82 (0.33 to 2.04)	0.68	0.60 (0.23 to 1.58)	0.30
	High Δ sPAP	1.03 (0.46 to 2.31)	0.93	1.00 (0.44 to 2.24)	0.99

Bold type indicates statistical significance (p<0.05).

ESE, exercise stress echocardiography; GLS, global longitudinal strain; LVEF, left ventricular ejection fraction; MAGGIC, Meta-Analysis Global Group In Chronic heart failure; MR, mitral regurgitation; sPAP, systolic pulmonary artery pressure.

### A subgroup of patients with moderate or greater MR

Since the degree of baseline MR may impact the results, we performed a sensitivity analysis in patients with moderate or greater MR at baseline (n=27). As summarised in online supplemental table 1, they were older and had significantly higher B-type natriuretic peptide and MAGGIC risk score, a larger chamber size, higher E/e’ and right atrial pressure, more impaired GLS in comparison with those with mild MR. However, the echocardiographic changes during each exercise were similar to the results in the entire cohort (online supplemental table 2) and the correlations between the changes observed during ergometer-ESE and during handgrip-ESE were weak to moderate (online supplemental figure 2). In addition, Kaplan-Meier curve analyses showed the same results that only the lack of improvement in GLS during ergometer-ESE, but no parameter during handgrip-ESE, was associated with the adverse events (online supplemental figure 3). Univariable Cox analysis also showed the same results as in the entire cohort (online supplemental table 3). Since the number of events (18, 67%) was too small in this subgroup, we did not try multivariable Cox analysis in the subgroup.

## Discussion

**Figure 3:**
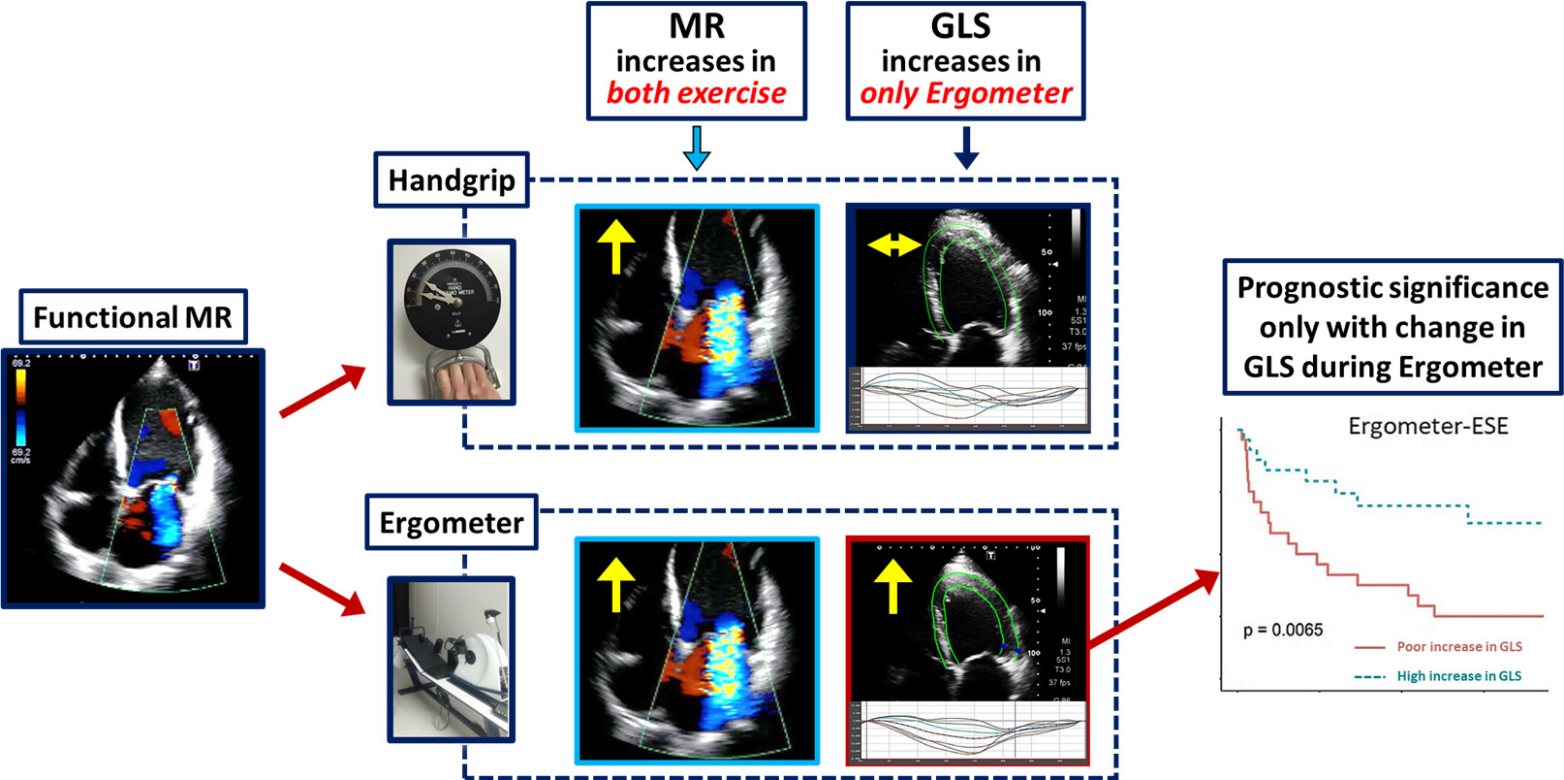
Outcomes of each exercise protocol. Ergometer-ESE resulted in an increase in both the systolic function and MR grade. Poor improvement in GLS was significantly associated with adverse events. Handgrip-ESE resulted in an increase in the MR grade without improvement in the GLS. None of the changes during handgrip-ESE were significantly associated with the patient outcomes. ESE, exercise stress echocardiography; GLS, global longitudinal strain; MR, mitral regurgitation.

This study reported the first direct comparison between two different types of exercise for ESE for patients with heart failure and secondary MR. The results showed that (1) with appropriate amount of exercise indicated by the changes in vital signs, MR was increased slightly but significantly greater during handgrip-ESE than ergometer-ESE, while other parameters were changed by less degrees during handgrip-ESE than during ergometer-ESE; (2) the correlation between the changes in the parameters during the two examinations was moderate at most, and factors associated with these changes differed between handgrip-ESE and ergometer-ESE, suggesting different underlying physiological changes during the two examinations; and (3) GLS improvement during ergometer-ESE, but not the change in MR, was associated with better clinical prognosis, while none of the echocardiographic changes observed during handgrip-ESE was prognostic, as summarised in figure 3. These findings were basically the same in the subgroup of the patients with moderate or greater MR at baseline. The study provided novel insights into the understanding of the physiological and prognostic implications of ESE and revealed the differences between the two exercises, thus raising a caution about handgrip-ESE as an alternative to ergometer-ESE.

### Physiological changes in cardiac function during exercise

The dynamic nature of secondary MR in various conditions is well known. Studies have reported that different kinds of stress conditions lead to different alterations in secondary MR:, such that increase in preload and afterload during the acute phase of heart failure commonly increases secondary MR,^7^ whereas pure improvement of cardiac function in response to dobutamine infusion reduces secondary MR.^26^ Although ergometer exercise is the most well-established exercise for ESE, elderly patients who are candidates for percutaneous valvular intervention are too frail to perform this exercise. Consequently, handgrip-ESE is used as an alternative, but the physiological implications of ergometer-ESE and handgrip-ESE are theoretically very different. While ergometer exercise is isotonic exercise in which large muscles, like the quadriceps and gluteal muscles, are in dynamic motion, resulting in volume overload due to increased venous return, handgrip exercise is isometric exercise in which the blood pressure and afterload are increased with a lesser increase in the LV preload.^27^ We observed a significant increase in the right atrial pressure and stroke volume during ergometer-ESE, while the increase in the right atrial pressure was minimum and the stroke volume did not change significantly during handgrip-ESE. Our exploratory analysis showed that factors associated with MR and GLS changes during handgrip-ESE were different from those noted during ergometer-ESE. Although various parameters were associated with ergometer-ESE, the main factors in handgrip-ESE were changes in blood pressure. These results suggested that the echocardiographic changes during ergometer-ESE were multifactorial complex outcomes of ergometer exercise, whereas the increase in the blood pressure and afterload was the main trigger for the changes observed during handgrip-ESE. However, since the exploratory analyses were based on speculations without detailed haemodynamic assessment using catheter examinations, further studies are warranted to elucidate the underlying mechanisms.

### Changes in the echocardiographic parameters and clinical outcomes

Although previous studies have reported the prognostic significance of the deterioration in MR, elevated PAP and lack of LV systolic function in primary MR,^10 11 28^ evidence for the significance of ESE in secondary MR is limited and inconsistent.^12 29 30^ Ennezat *et al* reported that the changes in the degree of secondary MR during ergometer exercise did not have prognostic implications for patients with severe LV dysfunction and mild-to-moderate MR.^29^ Here, we found that change in the GLS during ergometer-ESE, but not during handgrip-ESE, was the only parameter associated with all-cause mortality and cardiac hospitalisation. The changes in MR during either examination were not associated with adverse events.

The conflicting results from the recent COAPT and MITRA-FR trials have prompted an important discussion regarding the candidates appropriate for non-pharmacological intervention for secondary MR.^6 31^ Therefore, patients with severe MR and relatively preserved LV function might be the best candidates for such interventions, whereas those with mild secondary MR and extremely deteriorated LV might not benefit from such intervention and would not be good therapeutic targets. The results derived from the present population with a relatively milder degree of secondary MR and severe LV dysfunction supported the aforementioned findings. In such patients, the LV function was possibly a more important prognostic determinant than secondary MR. An important implication from our results was that even if MR grade increased during ESE, patients with moderate secondary MR may not be good candidates for non-pharmacological interventions.

### Limitations

Our study has several limitations. First, this was a single-centre study including a relatively small number of patients. Thus, the present results, especially the part regarding the clinical outcomes, should be considered as a hypothesis-generating pilot study. Further studies are warranted to confirm our findings. Next, treadmill exercise, one of the most popular types of ESE, was not tested in this study. However, since very quick echocardiographic scanning is required in treadmill ESE, it may not pragmatically be a good choice for valvular heart disease assessment. Another concern is that handgrip exercise tends to be insufficient especially in frail patients. However, in the present study, dedicated medical staff was observing the grip strength, confirming that the patient was appropriately keeping the grip strength during the exercise. In addition, the significant increase in MR during handgrip-ESE indicates that the strength of the exercise was substantial. Last, our study population included a substantial number of patients with mild secondary MR. Nevertheless, the subgroup analysis excluding patients with mild MR showed the same results as in the entire cohort. In addition, the importance of ESE only for patients with severe secondary MR at baseline is limited because most such patients are already symptomatic and do not require further stress tests for evaluation.

## Conclusions

Handgrip-ESE and ergometer-ESE have significantly different physiological and prognostic implications for patients with LV dysfunction and secondary MR. The pattern of cardiac haemodynamic changes during both types of ESE was different, and only improvement in the GLS during ergometer-ESE was associated with adverse events. These results suggest that handgrip-ESE may not be appropriate for risk assessment of patients with secondary MR, although handgrip-ESE is easier to perform. The type of exercise to be performed during ESE should be carefully selected.

## Data Availability

Data are available upon reasonable request directly to the authors.
