# Supplementary material for: Physiological and prognostic differences between types of exercise stress echocardiography for functional mitral regurgitation
Source: Open Heart. 2021 Apr 22;8(1):e001583. doi: 10.1136/openhrt-2021-001583 (PMC8070867; doi:10.1136/openhrt-2021-001583)

Supplementary Table 1. Characteristics in The Subgroups

Parameter	Moderate/Severe (n=27)	Mild (n=26)	p value
**Age, years old**	74 [65 - 80]	65 [53 - 72]	0.041
**Male, n (%)**	18 (66.7)	19 (73.1)	0.766
**Body mass index (kg/m2)**	21.6 [19.8 - 23.9]	23.3 [21.9 - 28.0]	0.067
**Systolic blood pressure, mmHg**	106 [97 - 125]	114 [105 - 120]	0.142
**Diastolic blood pressure, mmHg**	69 [60 - 74]	69 [65 - 77]	0.35
**Heart rate, /min**	70 [66 - 79]	68 [56 - 72]	0.06
**Past medical history**			
**Ischemic cardiomyopathy, n (%)**	12 (44.4)	12 (46.2)	1
**COPD, n (%)**	11 (40.7)	7 (26.9)	0.387
**Diabetes, n (%)**	17 (63.0)	14 (53.8)	0.583
**Heart Failure Severity**			
**NYHA class ≥ III, n (%)**	11 (40.7)	7 (26.9)	0.387
**B-type natriuretic peptide, pg/ml**	406 [254 - 535]	206 [143 - 351]	0.003
**MAGGIC score**	32 [28 - 35]	26 [19 - 31]	0.003
**Echocardiography**			
**IVSD, mm**	10 [8 - 11]	10 [9 - 11]	0.726
**LV diastolic diameter, mm**	63 [57 - 67]	56 [47 - 64]	0.021
**LV systolic diameter, mm**	56 [45 - 61]	44 [37 - 57]	0.029
**Left atrial diameter, mm**	47 [44 - 51]	40 [37 - 46]	0.006
**LV ejection fraction, %**	19 [15 - 32]	30 [19 - 43]	0.094
**Mitral E velocity, cm/s**	88 [69 - 110]	75 [59 - 97]	0.111
**Tissue Doppler e', cm/s**	3.9 [3.3 - 5.6]	5.1 [4.4 - 5.9]	0.046
**E / e'**	20.0 [14.3 - 32.2]	13.3 [11.5 - 18.9]	0.011
**Right atrial pressure, mmHg**	8 [8 - 8]	3 [3 - 8]	0.017
**Systolic PAP, mmHg**	36 [29 - 40]	31 [29 - 37]	0.19
**GLS, % (absolute value)**	6.9 [5.3 - 10.2]	11.4 [8.9 - 15.6]	0.004
**MR volume, ml**	25 [21 - 33]	14 [10 - 19]	<0.001
**Mitral EROA, cm2**	0.16 [0.13 - 0.23]	0.10 [0.07 - 0.11]	<0.001
**MR / LA area ratio**	0.27 [0.23 - 0.34]	0.12 [0.09 - 0.19]	<0.001
**Mitral valve tenting height, mm**	9.0 [8.0 - 11.7]	9.6 [7.6 - 10.7]	0.551
**MR grade, n (%)**			<0.001
**mild**	0 (0.0)	26 (100.0)	
**moderate**	19 (70.4)	0 (0.0)	
**moderate-severe**	7 (25.9)	0 (0.0)	
**severe**	1 (3.7)	0 (0.0)	

COPD, chronic obstructive pulmonary disease; NYHA, New York Heart Association; MAGGIC, Meta-Analysis Global Group In Chronic heart failure; LV, left ventricular; PAP, pulmonary artery pressure; GLS, global longitudinal strain; MR, mitral regurgitation; EROA, effective regurgitant orifice

Supplementary Table 2. Change by Each Exercise in The Significant MR Group

Parameter	Ergometer-ESE	Handgrip-ESE	p value (ergometer- vs. handgrip-ESE)
Δ Heart rate, /min	34 [21 - 47] **	10 [6 - 15] **	<0.001
Δ Systolic blood pressure, mmHg	29 [16 - 43] **	26 [7 - 39] **	0.06
Δ LV ejection fraction, %	6 [3 - 8] **	2 [1 - 4] **	<0.001
Δ Stroke volume, ml	10 [6 - 22] **	-1 [-8 - 3]	<0.001
Δ E / e'	1.5 [-1.2 - 5.3]	2.1 [-1.0 - 3.4]	0.46
Δ Right atrial pressure, mmHg	0 [0 - 6] *	0 [0 - 0]	0.006
Δ Systolic PAP, mmHg	24 [17 - 34] **	13 [10 - 19] **	0.001
Δ GLS, % (absolute value)	1.1 [0.7 - 2.2] **	0.0 [-1.1 - 0.8]	<0.001
Δ MR volume, ml	10 [4 - 16] **	14 [9 - 16] **	0.08
Δ LA / MR area	0.09 [0.02 - 0.12] *	0.07 [0.03 - 0.12] **	0.39
Δ MR grade	1 [0 - 1] *	1 [1 - 2] **	<0.001

Abbreviations are the same as in the table 1. * $p < 0.05$ vs. baseline; ** $p < 0.001$ vs. baseline. Paired t-tests were used for comparison except for MR grade which was compared using Wilcoxon signed rank test.

Supplementary Table 3. Cox Proportional Hazard Models for Adverse Events in The Significant MR Group

		Univariable	
Exercise type	Parameter	HR [95% CI]	p value
Ergometer-ESE	High Δ LVEF	0.58 [0.22 - 1.51]	0.26
	**High Δ GLS**	**0.32 [0.11 - 0.94]**	**0.037**
	High Δ MR volume	0.76 [0.30 - 1.94]	0.57
	High Δ sPAP	0.59 [0.21 - 1.67]	0.32
Handgrip-ESE	High Δ LVEF	1.21 [0.47 - 3.12]	0.70
	High Δ GLS	0.89 [0.35 - 2.27]	0.81
	High Δ MR volume	0.79 [0.31 - 2.00]	0.62
	High Δ sPAP	1.20 [0.44 - 3.32]	0.72

Abbreviations are the same as in previous tables

Supplementary Figures

Supplementary Figure 1. Bland Altman Plots

Plots show that there is no significant systemic errors between the handgrip and ergometer

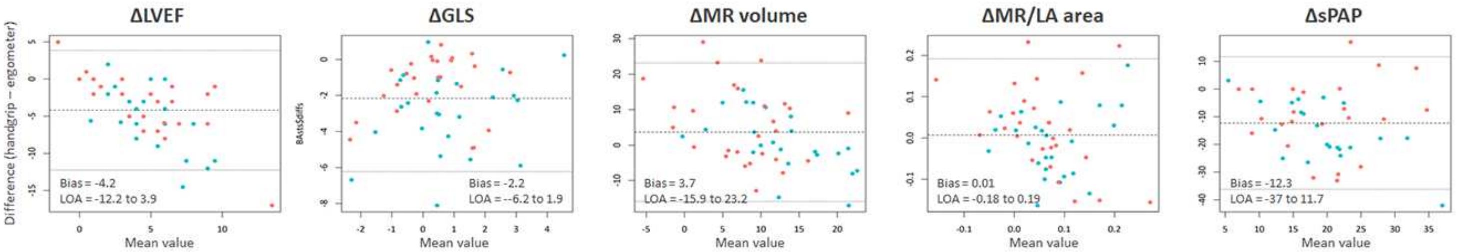

ESE.

Supplementary Figure 2. Correlation between the changes during handgrip- and ergometer-ESE in the subgroup

Similar to the entire cohort, changes in echocardiographic parameters were weak to moderate (r = 0.13 to 0.59), except for LVEF (r = 0.70) in the subgroup with patients with moderate or greater MR.

ESE, exercise stress echocardiography; GLS, global longitudinal strain; LA, left atrium; LVEF, left ventricular ejection fraction; MR, mitral regurgitation; PAP, systolic pulmonary artery pressure

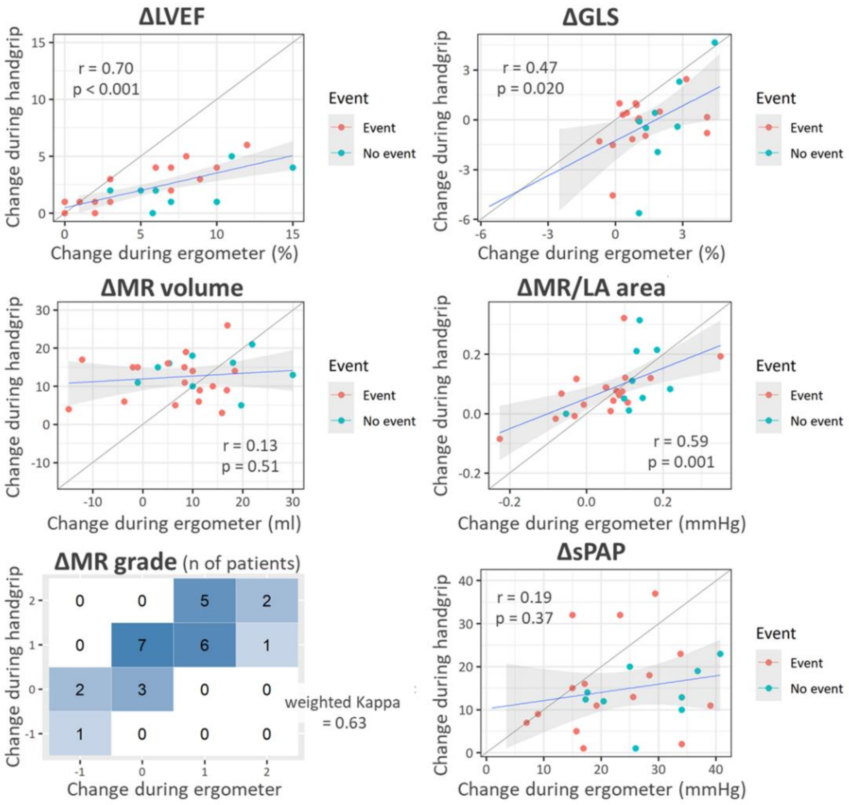

Supplementary Figure 3. Association of the changes during exercise with clinical outcomes in the subgroup

The prognostic results in the subgroup was the same as in the entire cohort; only the change in GLS during ergometer exercise was prognostic.

ESE, exercise stress echocardiography; GLS, global longitudinal strain; LVEF, left ventricular ejection fraction; MR, mitral regurgitation; PAP, systolic pulmonary artery pressure

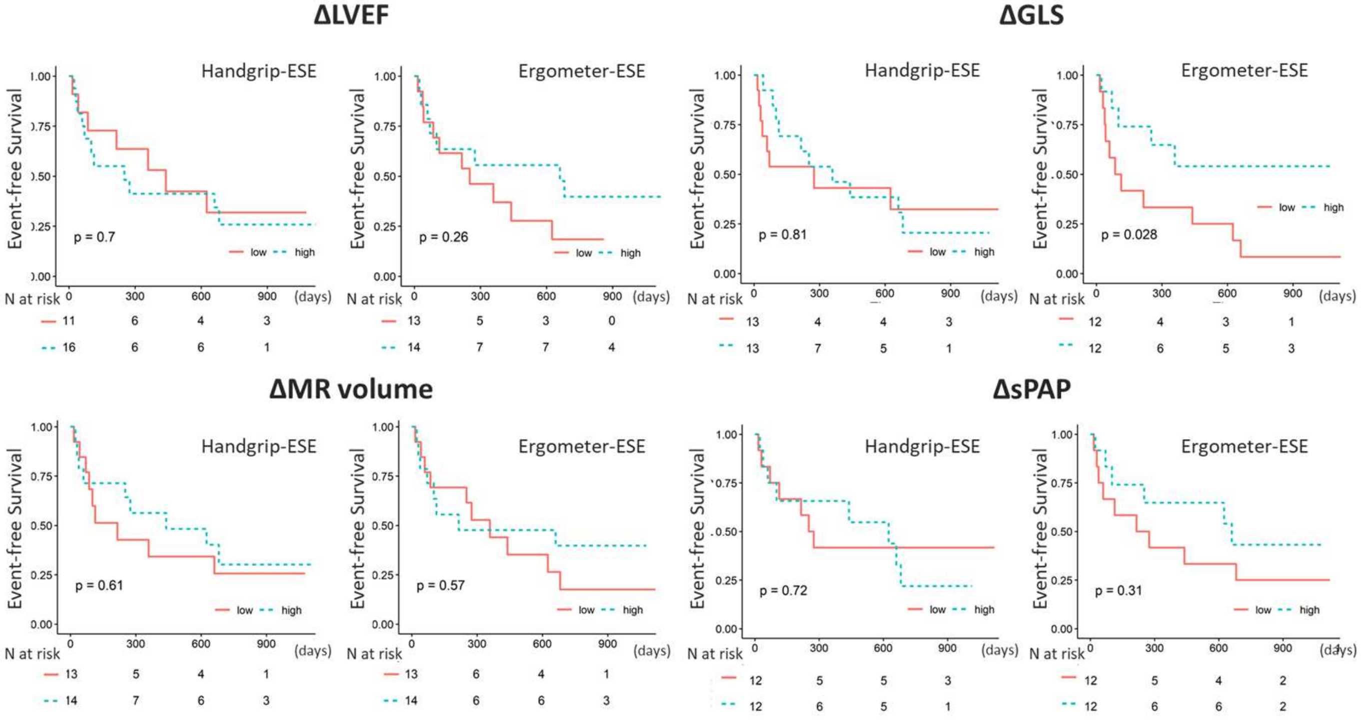

Supplement: Supplementary data [file openhrt-2021-001583supp001.pdf]
